# Expression and Impact of C1GalT1 in Cancer Development and Progression

**DOI:** 10.3390/cancers13246305

**Published:** 2021-12-15

**Authors:** Yangu Wan, Lu-Gang Yu

**Affiliations:** Department of Biochemistry and Systems Biology, Institute of Systems, Molecular and Integrative Biology, University of Liverpool, Liverpool L69 3GE, UK; Y.Wan10@liverpool.ac.uk

**Keywords:** C1GalT1, T synthesis, protein glycosylation, cancer, mucin, TF antigen, glycans, galectin-3

## Abstract

**Simple Summary:**

C1GalT1 is one of the enzymes that catalyze the addition of sugar residues to proteins (protein glycosylation). It specifically controls the synthesis and formation of a special disaccharide structure Galβ1,3GalNAcα-, which occurs predominately in cancer but rarely in normal cells. Recent studies have shown that C1GalT1 is overexpressed in many common cancers including colon, breast, gastric, lung, head and neck, pancreatic, esophageal, prostate, and hepatocellular cancer. C1GalT1 overexpression is also often associated with poorer prognosis and poorer patient survival. This review summarizes our current understanding of the expression of C1GalT1 in various cancers and discusses the impact of C1GalT change on cancer cell activities in cancer development and progression.

**Abstract:**

C1GalT1 (T-synthase) is one of the key glycosyltransferases in the biosynthesis of O-linked mucin-type glycans of glycoproteins. It controls the formation of Core-1 disaccharide Galβ1,3GalNAcα- (Thomsen–Friedenreich oncofetal antigen, T or TF antigen) and Core-1-associated carbohydrate structures. Recent studies have shown that C1GalT1 is overexpressed in many cancers of epithelial origin including colon, breast, gastric, head and neck, pancreatic, esophageal, prostate, and hepatocellular cancer. Overexpression of C1GalT1 is often seen to also be associated with poorer prognosis and poorer patient survival. Change of C1GalT1 expression causes glycosylation changes of many cell membrane glycoproteins including mucin proteins, growth factor receptors, adhesion molecules, and death receptors. This leads to alteration of the interactions of these cell surface molecules with their binding ligands, resulting in changes of cancer cell activity and behaviors. This review summarizes our current understanding of the expression of C1GalT1 in various cancers and discusses the impact of C1GalT change on cancer cell activities in cancer development and progression.

## 1. Introduction

Modification of proteins by carbohydrates is an important post-translational process. It assists adequate folding and stabilization of the newly synthesized proteins to become mature and functional molecules. Protein glycosylation can also directly regulate the activity and function of the proteins the carbohydrates are attached to. Glycosylation of proteins starting from the addition of N-acetyl-galactosamine (GalNAc) to serine and threonine residues of proteins is one of the major protein glycosylation types, the mucin-type O-glycosylation [1,2]. The biosynthesis of mucin-type O-glycosylation is controlled by sequential actions of an arrayof glycosyltransferases, each catalyzing the addition of unique monosaccharides to specific carbohydrate positions and sites [1,2,3].

The Core-1 β1,3galactosyltransferase (C1GalT1, T-synthase) is the glycosyltransferase that catalyzes the addition of galactose (Gal) to GalNAc to the formation of Galβ1,3GalNAcα-Ser/Thr (Thomsen–Friedenreich oncofetal antigen, T or TF antigen), the Core-1 structure of O-linked mucin-type glycans [4,5,6,7]. In normal cells, formation of this Core disaccharide structure is modified further by other carbohydrate residues to form longer and branched carbohydrate structures [3,6,7]. Alternatively, it can be modified by sialic acid residue to form sialyl-TF structure for chain termination [8,9,10]. In contrast to the existence of multiple glycosyltransferases for the addition of GalNAc to Ser/Thr for GalNAcα-Ser/Thr (Tn antigen) formation [11], C1GalT1 is known the only glycosyltransferase to catalyze the addition of Gal residue to GalNAcα-Ser/Thr [10,12]. C1GalT1 is encoded in the human genome by a single gene c1galt1 on chromosome 7q21.3 with 115.9 kb residues that consist of three exons of about 62kb [10,12]. C1GalT1 protein contains 363 amino acids and is a type-II transmembrane protein. Rate C1GalT1 is an 84/86KDa disulfide-bonded homodimer, but its active form is believed to be a 42/43 KDa monomeric protein [8,12].

**Figure 1:**
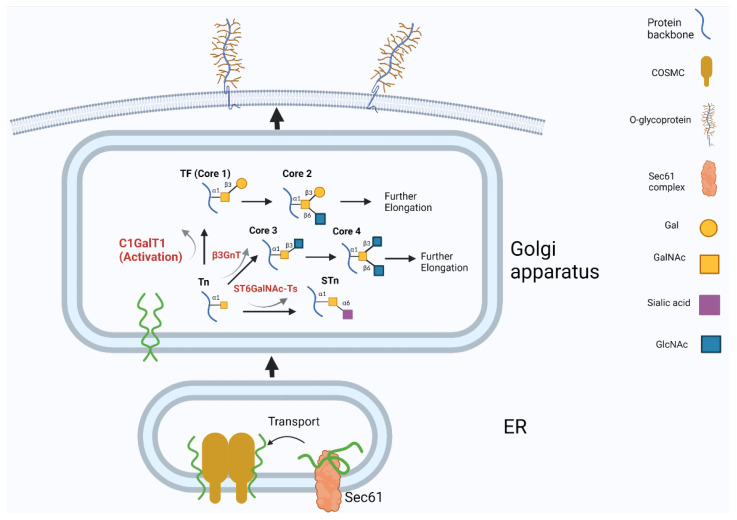
Regulation and action of C1GalT1 in biosynthesis of O-linked mucin-type glycans. Newly produced inactive C1GalT1 polypeptide is transported from the ribosome into ER by protein complex Sec61. C1GalT1 is converted in the ER into an active and dimerized form by molecular chaperon COSMC before it enters the Golgi apparatus. C1GalT1 competes with two other glycosyltransferase types (β3GnT6 and ST6GalNAC-I/II) in the Golgi apparatus to catalyze the addition of Gal to GalNAcα-Ser/Thr to the formation of Core-1 carbohydrate structure.

Activation of C1GalT enzyme activity requires the presence and function of a molecule chaperon COSMC located in the endoplasmic reticulum (ER) [8,13]. Like C1GalT1, COSMC is also a type-II transmembrane protein. It contains 318 amino acids with a short N-terminal in the cytoplasm and a large C-terminal in the ER [14,15,16]. COSMC binds to a Cosmc-binding region (CBR) on the newly produced inactive C1GalT1 polypeptide, which is transported from the ribosome across the ER membrane by a protein complex Sec61, to allow formation of a properly folded and dimerized form of C1GalT1 in the ER [14,17,18,19,20] (Figure 1). The adequately folded C1GalT1 is then released from COSMC and enters the Golgi apparatus to catalyze the addition of Gal to GalNAcα-Ser/Thr for Galβ1,3GalNAcα-Ser/Thr formation [18,19]. In the absence of COSMC, the inadequately folded C1GalT1 peptides are aggregated by other molecular chaperones, such as Grp78, that cleave the stem region of the C1GalT1 peptides [8,21]. The truncated C1GalT1 polypeptides are then translocated outside of ER by HRD 1 complex, ubiquitinylated and degraded by the 26S proteasome system [8,16,22].

## 2. C1galt1 Is Overexpressed in Many Epithelial Cancers and Is Associated with Poorer Prognosis and Poorer Patient Survival

Overexpression of C1GalT1 occurs in various cancer types of epithelial origin. Significantly higher C1GalT1 expression at protein or mRNA levels is seen in lung [23], colon [24,25], breast [26,27], gastric [28,29], head and neck [30], pancreatic [31], esophageal [32], prostate [33], ovarian [34], and hepatocellular cancers [35] (Table 1). Overexpression of C1GalT1 is also associated with poorer prognosis and low survival in patients of colon [24], breast [26], esophageal and laryngeal [32,36], head and neck [30], lung [23], and prostate cancers [33]. The association of high C1GalT1 expression with poorer prognosis in breast cancer patients can be further enhanced by the presence of high levels of the polypeptide N-acetyl-galactosaminyltransferase (GALNT) family members GALNT1 or GALNT8 [37]. High C1GalT expression correlates with increasing cancer stages in prostate cancer [33] and with increasing malignant potency in breast cancer [26]. Patients with lower C1GalT1 expression were reported to survive 20% better over a 100-month period than those with higher C1GalT1 expression in breast cancer [37]. In colorectal cancer, patients with higher C1GalT1 expression were shown to have 20% poorer survival over 2000 days in comparison to those with lower C1GalT1 expression [24]. In hepatocellular cancer, patients with low C1GalT1 expression had a doubled survival rate compared to those with high C1GalT expression over 60 months [38]. In gastric cancer, increasing expression of C1GalT1 was associated with decreasing survival of patients over 60 months [28,39]. Patients of pancreatic cancer with high C1GalT1 expression were shown to survive 18 months less compared to those with low C1GalT1 expression within 4 years [31]. It is clear that overexpression of C1GalT1 is a common feature of epithelial cancers and is associated with worse prognosis and low survival.

Inoculation of C1GalT knockdown human colon cancer SW620 cells into NOD/SCID mice was shown to develop substantially smaller and lighter tumors than those inoculated with control SW620 cells in the animals [24]. More lung metastasis was observed in mice injected with higher C1GalT-expressing SW480 cells than those injected with lower C1GalT-expressing SW620 cells [24]. Similar results were also seen in mice inoculated with gastric, hepatocellular, head and neck, lung, and prostate cancer cells [23,28,30,33,35,38]. These studies indicate that overexpression of C1GalT1 in cancer promote cancer development, progression, and metastasis.

It should be mentioned that, although mice orthotopically injected with C1GalT knockdown pancreatic cancer HPAC and HPAF-II cells grew smaller tumors than those injected with control cells [31], c1galt1 knockout mice created by crossing c1galt1 floxed (C1galt1^loxp/loxp^) mice with Kras^G12D/+^; Trp53^R172H/+^; Pdx1-Cre (KPC) mice showed lower survival than KPC mice [40]. These engineered mice were also seen to develop pancreatic neoplastic more easily and were malignant to pancreatic ductal adenocarcinoma (PDAC) faster than KPC mice [40]. The discrepancy reported in these two studies is likely related to the different immune responses of the animals as one study used immunocompromised NOD/SCID mice while the other used genetically engineered mice. It is also possible that the strong effects of Kras and Trp53 genes may have overshadowed the influence C1GalT in the latter study where engineered mice were used [40].

### 2.1. Possible Mechanisms of C1GalT1 Overexpression in Cancer

The mechanism of C1GalT1 overexpression in cancer remains largely unknown. A recent study has suggested SP1 as a potential transcription factor of C1GalT1 expression [29]. SP1 is a well-characterized transcriptional activator [43,44]. It activates the transcription of many cellular genes that contain CG-rich Sp-binding sites in their promoters. It is involved in a variety of biological processes including cell growth and carcinogenesis [45,46]. Two potential SP1-binding sites (−676_−666bp; −67_−57bp) were speculated on the c1galt1 promotor region [29]. SP1 knockdown was shown to decrease C1GalT1 expression while SP1 overexpression increase C1GalT1 expression in gastric cancer cells [29]. As overexpression of SP1 occurs in many cancers [47], it is likely that SP1 overexpression makes an important contribution to the increased C1GalT1 expression in cancer.

It has been suggested that microRNA (miRNA) may also regulate C1GalT1 expression [23]. Fourteen miRNAs were predicted using Algorithms prediction tool, TargetScan, to modulate C1GalT1 expression. Binding of miR-181d-5q to the 3′UTR site of C1GalT1 indeed decreased C1GalT1 expression in lung cancer cells [23], while binding of miR-152 to the 3′UTR site on C1GalT1 reduced C1GalT1 expression in gastric cancer cells [29]. Thus, regulation of C1GalT1 overexpression in cancer occurs at least at the transcription level and likely involves multiple regulators.

### 2.2. C1GalT Overexpression Increases the Occurrence of TF Antigen on Cancer Cell Surface

C1GalT1 catalyzes the addition of Gal to the GalNAc residue on GalNAcα-Ser/Thr for the formation of Core-1-associated carbohydrate structures. Modification of the GalNAc residue by C1GalT1 is competed for by two other glycosyltransferase types for the formation of different carbohydrate structures, i.e., by β3GnT6 for Core-3-related carbohydrate structure formation of chain elongation and by ST6GalNAC-I/II for sialyl-GalNAcα-Ser/Thr (STn) formation of chain termination [2,4,15,48] (Figure 1). Overexpression of C1GalT1 expression in cancer cells disrupts the balance of competition among these glycosyltransferases and favors the formation of Core-1-associated carbohydrate structures, such as Galβ1,3GalNAcα-Ser/Thr [48,49,50].

The unsubstituted Galβ1,3GalNAcα-Ser/Thr is an oncofetal carbohydrate antigen that is seen in >90% cancer of epithelial origin but rarely in normal epithelia [11,51,52,53,54]. The appearance of TF antigen itself in cancer is an indication of poor prognosis and poor survival of patients [16,55,56]. Several cell membrane glycoproteins, such as MUC1 [57], CD44v6 [51], and integrins [58,59,60], are known to carry unsubstituted TF antigen in various cancers, such as colon [24], breast [26], and pancreatic [31] cancer. Peanut agglutinin-affinity purification followed by proteomic analysis suggests that cell membrane CD34, CD59, and CD133 may also carry unsubstituted TF structures in prostate and breast cancer cells [58,60,61].

The increased appearance of unsubstituted TF antigen by cancer cells can lead to increased interaction of the tumor cells with galactoside-binding galectins and influences cancer progression and metastasis [62,63,64,65]. Interaction of TF on mucin protein MUC1 on tumor cells with galectin-3 increases tumor cell–cell homotypic aggregation and promotes tumor cell emboli formation and survival in the circulation [66,67]. Interaction of TF/MUC1 with galectin-3 also enhances tumor cell–endothelial heterotypic adhesion in circulating tumor cell extravasation [66,67]. Overexpression of C1GalT1 in cancer cells, which increases cellular TF expression, thus aids circulating tumor cell hematogenous dissemination in metastasis by enhancing tumor cell interaction with galectins.

### 2.3. Change of C1GalT1 Expression Alters Glycosylation of Cell Membrane Mucin Proteins and Their Interaction with Partner Molecules

Most cell membrane proteins are glycoproteins [58,68]. Change of C1GalT1 expression or activity in cancer often alters the O-linked carbohydrate structures on these cell membrane glycoproteins and changes their activity [69].

Mucin proteins are important components of the cell membrane of epithelial cells. They provide physical protection to the epithelium from damage by harmful substances (e.g., toxin in the gut) and also mediate outside-in signal transduction in cells’ response to environment changes [3,54]. Mucin proteins are heavily glycosylated with O-linked carbohydrates, which typically make up 30~50% of the mucin protein molecular weights [70,71]. Glycosylation of mucin proteins is therefore particularly prone to C1GalT1 expression changes in cancer. Indeed, substantial expressions of TF antigen are seen on mucin protein MUC1, MUC4, and MUC16 in colon, breast, and pancreatic cancer than in their normal counterparts [42,57,72]. Suppression of C1GalT1 expression in pancreatic ductal adenocarcinoma (PDAC) reduced MUC1 glycosylation and led to the appearance of shorter chain carbohydrate structures, such as Tn [40]. Overexpression of C1GalT in breast cancer cells increased the MUC1 molecular size, an indication of increased MUC1 O-glycosylation [26]. Impairing C1GalT1 activity by knockdown of its molecule chaperone COSMC reduced TF occurrence on mucin protein MUC5AC in BEAS-2B lung epithelial cells [42].

Change of mucin protein glycosylation in response to C1GalT1 alteration in cancer cells can lead to a significant change of the mucin protein communication with adjacent molecules and influence the activity and function of these molecules. As discussed above, C1GalT1-associated TF occurrence on MUC1 on cancer cells increases cancer cell interaction with galectins and promotes cancer cell progression and metastasis. It has been reported that interaction of cancer-associated TF on MUC1 with galectin-3 increases the interaction of MUC1 with EGFR on the cell surface [73]. This causes a significant increase of EGFR dimerization, activation, and signaling in the EGFR response to EGF binding in human breast and colon cancer cells [73]. Overexpression of C1GalT1 by transfection in breast cancer MCF-7 cells increased the interaction of the MUC1 C-terminal with β-catenin, causing increased cancer cell migration and invasion in vitro and increased tumor growth in mice [26]. Suppression of C1GalT1 expression by shRNA in PDAC cells reduced MUC16 O-glycosylation and increased MUC16 interaction with receptor tyrosine kinases (RTKs), such as EGFR and HER2 and integrin α4, on the cell membrane. This led to increased activation of EGFR and integrin on the cell surface and cell proliferation [40]. Thus, C1GalT overexpression in cancer cells can cause substantial glycosylation changes of cell membrane mucin proteins. These glycosylation changes can alter mucin protein interaction with their partners and lead to significant changes of the function and behaviors of these molecules in cancer.

### 2.4. Change of C1GalT1 Expression Alters the Glycosylation and Function of Receptor Tyrosine Kinases

RTKs are families of high-affinity cell membrane receptors for growth factors and cytokines and are critical regulators of cancer development [74,75]. RTKs are Class I transmembrane glycoproteins, each composing of an extracellular, a transmembrane, and an intracellular domain [75,76,77,78]. The RTK intracellular domains often contain multiple phosphorylation sites and can be modified by phosphorylation in cell response to ligand binding for receptor activation [76,79,80]. The extracellular domains of RTKs contain the growth factor/cytokine-binding sites and mostly carry both N- and O-linked carbohydrate structures [80,81]. A number of studies have reported that cancer-associated RTKs, such as EGFRs, fibroblast growth factor receptor-2 (FGFR2), EphA2, and hepatocyte growth factor receptor (HGFR), all carry truncated O-glycans in their extracellular domains [24,28,30,38]. Unsubstituted TF antigen occurs on FGFR2 in human colon cancer HCT116, SW620, and SW580 cells [24]. Transfection of human colon cancer HCT116 and SW480 cells with C1GalT1 reduced the binding of Tn-binding lectin VVA to FGFR2 while knockdown C1GalT1 increased VVA binding to FGFR2 (indications of increased and reduced FGFR2 O-glycosylation, respectively) in the cells [24]. Overexpression of C1GalT1 in colon cancer cells changed the O-glycan structures of FGFR2, which enhanced FGFR2 interaction with bFGF- and bFGF-mediated malignant phenotypes [24,38].

In head and neck squamous cell carcinoma (HNSCC), suppressing C1GalT1 expression by siRNA shortened the EGFR O-linked sugar chains and decreased the affinity of EGF binding to EGFR [30]. In gastric cancer AGS cells, suppression of C1GalT1 expression altered EphA2 O-glycosylation and reduced the EphA2 binding by its ligand Ephrin A1, resulting in deceased EphA2 phosphorylation and reduced cell migration and invasion [28]. Enhanced C1GalT1 expression by transfection reduced the occurrence of Tn antigen on RTK member MET and inhibited HGF-induced MET activation and viability of HA22T and PLC5 hepatocellular cancer cells [34]. As activation of RTKs is often essential in tumor cell growth and development, overexpression of C1GalT1, which changes RTK glycosylation and their sensitivity to ligand binding, is therefore likely involved in regulating RTK-mediated activity in cancer development.

### 2.5. Change of C1GalT1 Expression Alters the Glycosylation and Function of Cell Surface Integrins

Integrins are an important family of cell adhesion molecules in cell–cell and cell–environment communication. Each integrin molecule is a ubiquitous heterodimer, composed of one α- and one β- subunit [82,83]. At least 24 integrin heterodimers are known to date, made from 18 α- and 8 β- subunits [84]. Interaction of integrins with components of extracellular matrix (ECM) is directly involved in tumor cell adhesion, migration, and invasion in epithelial cancer progression [82,84,85,86]. Integrins are glycoproteins and various integrin forms have been reported to carry truncated O-glycans, such as TF structure, in different cancers. Integrins-α2, -α6, and -β1 were shown to express unsubstituted TF in prostate cancer while β1 integrin was reported to carry unsubstituted TF in laryngeal cancer [35,36,58]. Overexpression of C1GalT in pancreatic cancer reduced the appearance of the TF precursor structure Tn on integrin-αV and -α5 whereas knockdown of C1GalT1 in T3M4 PDAC cells increased Tn occurrence on mucin protein MUC16 and increased phosphorylation of integrin-α4 [31,40]. C1GalT1 overexpression in hepatocellular cancer HCC36 cells by transfection increased TF occurrence on integrin-β1, whereas knockdown of C1GalT1 by shRNA in HA22T hepatocellular cancer cells suppressed the TF expression on integrin-β1 [35]. Overexpression of C1GalT1 in hepatocellular cancer cells increased the occurrence of TF and STn structures on integrin-β1 and increased integrin activation and FAK signaling, leading to increased cell adhesion, migration, and invasion [35]. Mice injected with C1GalT1-overexpressing hepatocellular cancer cells produced more metastasis nudes on the animal lungs in comparison to mice injected by C1GalT1 knockdown cells [35]. These studies indicate that change of integrin O-glycosylation in response to C1GalT expression change can have a significant impact on integrin signaling and integrin-mediated cell adhesion in cancer progression.

It was reported that suppressing the expression of C1GalT1 by siRNA in Eca109 esophageal cancer cells reduced the occurrence of TF on integrin-β1 and decreased integrin downstream FAK and Akt signaling and cell adhesion [32]. This led to a reduction in cell tolerance to X-ray radiation [32]. Higher resistant to X-ray treatment was also seen in the higher C1GalT1-expressing Hep-2max cells, whose integrin-β1 carry more TF carbohydrate structures, than the low C1GalT1-expressing Hep-2min laryngeal cancer cells [36]. These studies indicate that change of integrin glycosylation in response to C1GalT1 expression change in cancer cells may modulate the sensitivity of tumor cells to radiotherapeutic treatment.

### 2.6. C1GalT1 Activity Regulates the Glycosylation and Function of Cell Surface Death Receptors

There are reports that C1GalT activity change regulates the carbohydrate structures of cell surface death receptors and affects their action in response to death stimuli. COSMC mutation in immortalized human T lymphocyte Jurkat cells increased the appearance of shorter carbohydrate structures Tn/STn on death receptors DR4 and DR5 and reduced cell death in cell response to tumor necrosis factor-related apoptosis-inducing ligand (TRAIL) [87]. COSMC-mediated glycosylation changes on death receptors DR4 and DR5 were seen to reduce homo-oligomer formation of these death receptors but increase the hetero-oligomers formation of the death receptors with decoy proteins, such as DcR2. As these decay proteins lack the intracellular death domain that is required by downstream apoptosis signaling, the death signaling was not able to pass on, hence cell death was prevented [87]. In breast cancer MDA-MB-231 and MCF-7cells, transfection of COSMC reduced cellular Tn and STn occurrence and increased cell apoptosis [88]. These studies indicate that overexpression of C1GalT1 expression in cancer cells affects the expression and length of O-linked carbohydrate structures on cell death receptors and influences their response to cell death stimuli in cancer development.

## 3. Conclusions Remarks

There is solid evidence in the literature that overexpression of C1GalT1 commonly occurs in epithelial cancers and is associated with poor prognosis and poor patient survival. Change of C1GalT1 expression in cancer leads to alteration of O-linked carbohydrate structures on many cell membrane glycoproteins, such as mucin proteins, growth factor receptors, adhesion molecules, and death receptors. Glycosylation changes of these cell membrane glycoproteins alter the interaction of these molecules with their binding ligands, leading to changes of their activity in the regulation of cancer development and progression. As C1GalT1 is a glycosyltransferase that controls the biosynthesis of a core glycan structure in cells, the reported glycosylation changes and impact on these cell membrane glycoproteins likely represent only the tip of an iceberg and many other cellular glycoproteins are most likely also affected by C1GalT1 overexpression in cancer. These glycosylation changes may also have a significant influence on tumor cell behaviors in various stages of cancer development, progression, and metastasis. Targeting the activity of C1GalT1 or its regulators may have therapeutic potential in the development of novel cancer treatment strategies.

## Figures and Tables

**Table 1:** C1GalT1 expression in various cancer and its impact on cancer cell behaviors and patient survival.

Cancer Type	C1GalT1 Expression	Effects on Cancer Cell Behaviors	Effects on Cell Signaling	Patient Survival	References
Colon	Increased	proliferation↑; migration↑; invasion↑; sphere formation↑	EGF-ERK; FGFR2; PI3K-Akt	lower	[24,25]
Breast	Increased	viability↑; proliferation↑; migration↑; invasion↑	CD44 inducing ERK-MAPK, p38/SAPKs, JNK;	lower	[26,27]
Pancreatic	Increased	viability↑; migration↑; invasion↑;	Integrin α5-FAk; Integrin αV-FAk Nucleoin, Grp-78, α-enolase; annexin A2; MUC16 inducing p-EGFR, α4 integrin and p-HER2	lower	[31]
	Decreased	migration↓; proliferation↓			[40,41]
Hepatocellular	Increased	adhesion↑; migration↑; invasion↑	HCF/MET; β1 integrin-FAK	lower	[35,38]
Gastric	Increased	viability↑; proliferation↑; migration↑; invasion↑	ephrinA1-EphA2 Integrin α5-FAk; PI3K-AKt	lower	[28,29]
Head and neck	Increased	viability↑; migration↑; invasion↑	EGF-EGFR	lower	[30]
Oesophageal	Increased	radiotherapy resistance↑	β1 integrin-FAk	lower, and increased resistance to radiotherapy	[32]
Laryngeal	Increased	radiotherapy resistance↑	β1 integrin-FAk	lower, and increased resistance to radiotherapy	[36]
Lung	Increased	proliferation↑; migration↑; colony formation↑	Neutrophil Elastase (NE) via MUC5AC; PI3K, EGFR, Ras, p85; RAC1	lower	[23,42]
Prostate	Increased	colony formation↑; sphere formation↑; proliferation↑	Co-effect with galectin-4 to HER2	lower	[33]
Ovarian	Increased	proliferation↑; migration↑; sphere formation↑	CD133, CD24, Oct4, Nanog and SNAI2	lower	[34]

## Abbreviations

C1GalT1	Core-1 β1:3galactosyltransferase
CBR	COSMC-binding region
ECM	extracellular matrix
EGFR	Epidermal growth factor receptor
ER	Endoplasmic reticulum
FGFR2	Fibroblast growth factor receptor-2
Gal	galactose
GalNAc	N-acetyl-galactosamine
GALNT	N-acetyl-galactosaminyltransferase
HER2	human epidermal growth factor receptor-2
HGFR	hepatocyte growth factor receptor
HNSCC	head and neck squamous cell carcinoma
PDAC	pancreatic ductal adenocarcinoma
RTK	receptor tyrosine kinase
STn	sialyl-GalNAcα-Ser/Thr
TF	Galβ1,3GalNAcα-ser/Thr (Thomsen–Friedenreich antigen)
Tn	GalNAcα-Ser/Thr
TRAIL	tumor necrosis factor-related apoptosis inducing ligand
VEGFR2	Vascular endothelial growth factor-2
VVA	Vicia Villosa lectin
